# Clinical Warburg effect in lymphoma patients admitted to intensive care unit

**DOI:** 10.1186/s13613-023-01192-z

**Published:** 2023-10-05

**Authors:** Anis Chaba, Sofiane Fodil, Virginie Lemiale, Eric Mariotte, Sandrine Valade, Elie Azoulay, Lara Zafrani

**Affiliations:** 1 Medical Intensive Care Unit, Saint-Louis University Hospital, Assistance Publique-Hôpitaux de Paris (AP-HP), 1 Avenue Claude Vellefaux, 75010 Paris, France; 2 Department of Hematology, Saint-Louis University Hospital, Assistance Publique-Hôpitaux de Paris (AP-HP), Paris, France; 3 University Paris Cité, Paris, France; 4 INSERM, UMR 944, University Paris Cité, Paris, France

**Keywords:** Warburg effect, Lactate, Lymphoma, Intensive care unit

## Abstract

**Background:**

The Warburg effect, characterized by elevated lactate levels without tissue hypoxia or shock, has been described in patients with aggressive lymphoproliferative malignancies. However, the clinical characteristics and long-term outcomes in this population remain poorly understood.

**Methods:**

We retrospectively analyzed 135 patients with aggressive lymphoproliferative malignancies admitted to the ICU between January 2017 and December 2022. Patients were classified into three groups: Clinical Warburg Effect (CWE), No Warburg with High Lactate level (NW-HL), and No Warburg with Normal Lactate level (NW-NL). Clinical characteristics and outcomes were compared between the groups and factors associated with 1-year mortality and CWE were identified using multivariable analyses.

**Results:**

Of the 135 patients, 46 (34%) had a CWE. This group had a higher proportion of Burkitt and T cell lymphomas, greater tumor burden, and more frequent bone and cerebral involvement than the other groups. At 1 year, 72 patients (53%) died, with significantly higher mortality in the CWE and NW-HL groups (70% each) than in the NW-NL group (38%). Factors independently associated with 1-year mortality were age [HR = 1.02 CI 95% (1.00–1.04)], total SOFA score at admission [HR = 1.19 CI 95% (1.12–1.25)], and CWE [HR = 3.87 CI 95% (2.13–7.02)]. The main factors associated with the CWE were tumor lysis syndrome [OR = 2.84 CI 95% (1.14–7.42)], bone involvement of the underlying malignancy [OR = 3.58 CI 95% (1.02–12.91)], the total SOFA score at admission [OR = 0.81 CI 95% (0.69–0.91)] and hypoglycemia at admission [OR = 14.90 CI 95% (5.42–47.18)].

**Conclusion:**

CWE is associated with a higher tumor burden and increased 1-year mortality compared to patients without this condition. Our findings underscore the importance of recognizing patients with CWE as a high-risk cohort, as their outcomes closely resemble those of individuals with lymphoma and shock, despite not requiring advanced organ support. Clinicians should recognize the urgency of managing these patients and consider early intervention to improve their prognosis.

**Supplementary Information:**

The online version contains supplementary material available at 10.1186/s13613-023-01192-z.

## Introduction

A century ago, in 1923, Otto. H Warburg raised the hypothesis that malignant cells may preferentially use the lactic fermentation pathway even in the presence of oxygen [1]. He suggested that this phenomenon could be attributed to an irreversible respiratory injury that may also have been the origin of cancer cells [2]. Based on Warburg’s work, several authors have reported that while this phenomenon appears to be a common feature of many types of tumors, there is clear evidence that glycolysis is enhanced without significant mitochondrial dysfunction [3–7]. These results marked a turning point in the history of the Warburg effect, as they shifted the paradigm towards the idea that the effect is due to “dysregulation of glycolysis” rather than “damage to respiration” [8]. Therefore, the upregulation of glucose conversion to lactate, even in the presence of functional mitochondria, does not appear to be a response to the hypoxic tumor microenvironment. In contrast, this phenomenon appears to be an acquired hallmark that emerges early in oncogenesis and may even characterize the process of uncontrolled growth [9, 10]. Based on these studies, we now have a more precise understanding of the intricate mechanisms underlying the Warburg effect historically classified as a B lactic acidosis [7, 11–18]. However, despite increasing pre-clinical knowledge about the Warburg effect, clinical data focusing on these patients remain scarce and rely only on case reports. Thus, we conducted a retrospective study to assess the long-term prognosis of patients with aggressive lymphoproliferative malignancy and identify the main clinical factors associated with the clinical Warburg effect.

## Methods

We conducted a retrospective single-center cohort study in a university hospital Intensive Care Unit (ICU). This study was approved by an Institutional Review Board (IRB) (“Comité d’Evaluation de l’Ethique des projets de Recherche Biomédicale Paris Nord” (IRB 00006477- of Paris Cité University). According to the French regulation, the need for informed consent was waived. However, patients were informed that their data might be used for research purposes and none of them refused. The study was conducted following the Declaration of Helsinki principles.

### Definitions

**Fig. 1:**
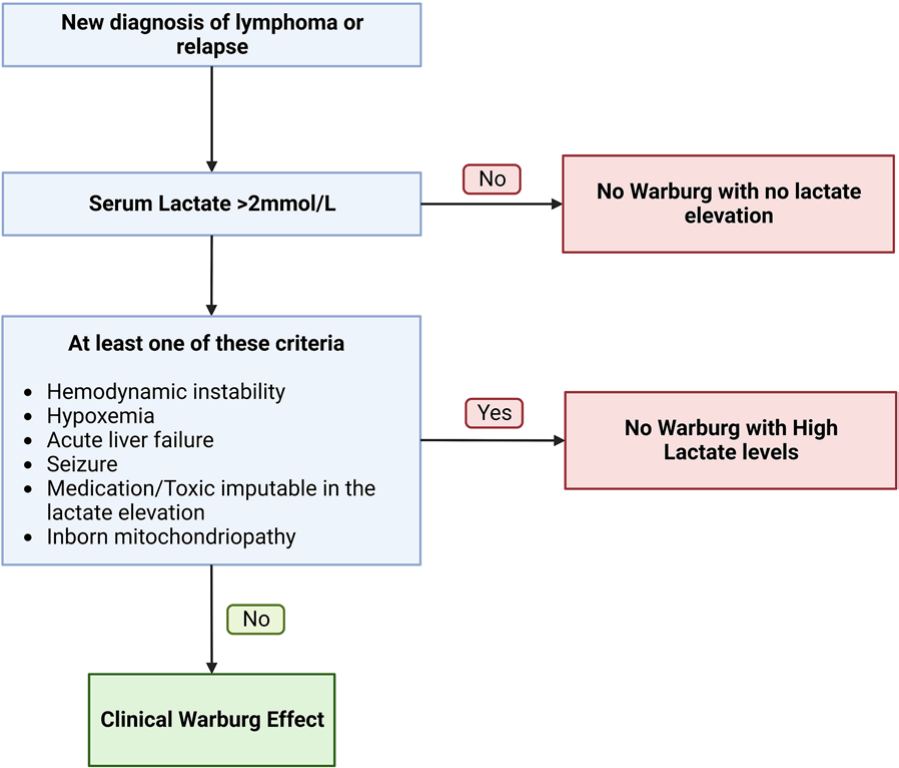
Clinical Warburg effect algorithm

We defined as Clinical Warburg Effect (CWE) the group of patients with lactatemia > 2 mmol/L without hemodynamic instability (mean arterial blood pressure < 65 mmHg or vasopressor requirement), severe hypoxemia (PaO_2_:FiO_2_ ≤ 300 mmHg), tissue ischemia (any documentation of macrovessel occlusion), acute liver failure, medication, or toxic potentially involved in type B2 lactic acidosis or inborn mitochondrial disorders. We divided the remaining patients into two groups: (1) No Warburg with No Lactate elevation (NW-NL) and No Warburg with High Lactate level (NW-HL) (Fig. 1). Acute liver failure was defined following the 2017 EASL practical guidelines [19]. We classified patients receiving metformin in their medication as non-Warburg patients. Hypoglycemia was defined as a glucose level at ICU admission < 5.5 mmol/L. The Sequential Organ Failure Assessment (SOFA) score and the Simplified Acute Physiology Score (SAPS II) score were also recorded, as previously defined [20–22]. Biological and clinical diagnosis of tumor lysis syndrome was established according to consensus definition [23]. Finally, hemophagocytic lymphohistiocytosis (HLH) diagnosis was established according to the classification developed by the Histiocyte Society and confirmed by attending hematologists and intensivists [24, 25].

### Study population

We screened from each ICU reports of Saint-Louis University Hospital from January 2017 to December 2022 for patients admitted with newly diagnosed aggressive lymphoproliferation or a new diagnosis of relapse. The screening method is detailed in Additional file 1. Patients were admitted from outside the hospital or from one of the eight hematology wards within the hospital. The Saint-Louis University Hospital is a 700-bed public hospital with 483 beds for patients with hematological malignancies and solid cancers. The ICU is a 20-bed medical unit that admits 1050 patients annually, of whom about one-third have hematologic malignancies. Information on the organization of our ICU and criteria for ICU admission have been published elsewhere [26]. Of note, our center advocates for the early admission of critically ill cancer patients [27]. This approach is especially relevant for patients with a newly diagnosed lymphoma or leukemia. For selected high-risk patients who are about to undergo their first line of chemotherapy, early ICU admission is then often considered. This approach often leads to admissions for patients with CWE who require the initiation of high-risk chemotherapy, even in cases where there is no organ failure upon admission. ICU admission policies remained unchanged throughout the study period.

### Objectives and outcomes

The primary objective was to identify factors associated with mortality at 12 months. The secondary objective was to identify factors associated with the CWE.

### Statistical analyses

Baseline characteristics are reported as absolute values with percentages for categorical variables or median with the interquartile interval for quantitative variables. Logistic regression and Cox model were used to assess the risk factors of CWE and 1-year mortality, respectively. To further assess the influence of CWE on mortality, we performed an overlap propensity score weighting analysis [28–30] and a complex bootstrap resampling. Further details about the statistical analysis are reported in Additional file 1.

### Missing data management

All data regarding the primary outcome were retrieved. Patients with missing lactate concentration at admission were not included in the primary analysis but are described in the (Additional file 1: Table S1).

## Results

### Patients’ characteristics

**Fig. 2:**
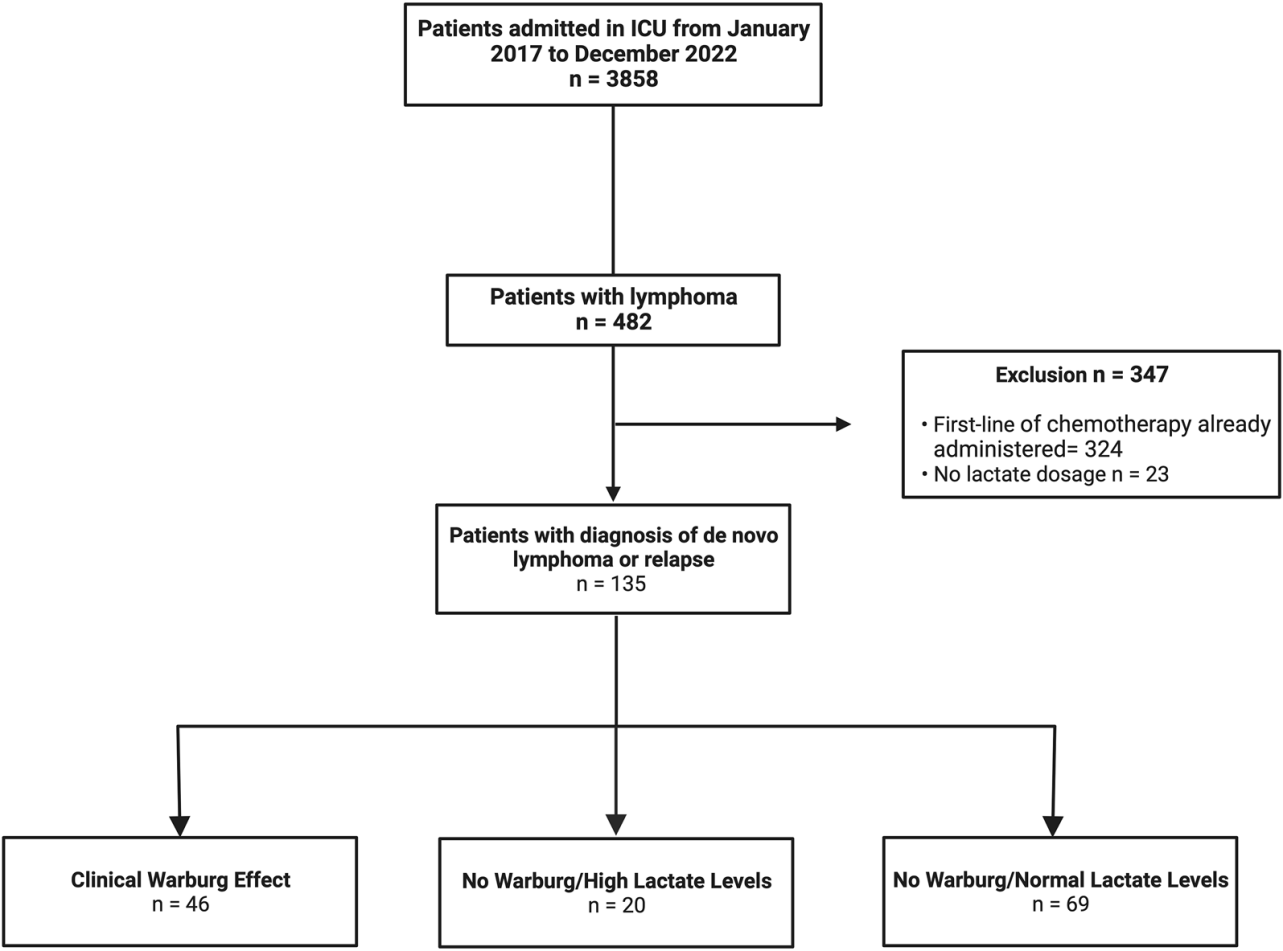
Flowchart

From January 2017 to December 2022, we screened 3858 medical reports of patients admitted to the ICU. Among them, 483 had an aggressive lymphoproliferative malignancy. We excluded 323 patients who had already received first-line chemotherapy treatment and 23 without lactate measurement. We included 135 patients diagnosed with de novo lymphoproliferative disease or a diagnosis of relapse. Among them, 46 patients fulfilled the CWE criteria, 20 were classified as No Warburg with High Lactate level (NW-HL) and 69 as No Warburg with Normal Lactate level (NW-NL) (Fig. 2).

**Fig. 3:**
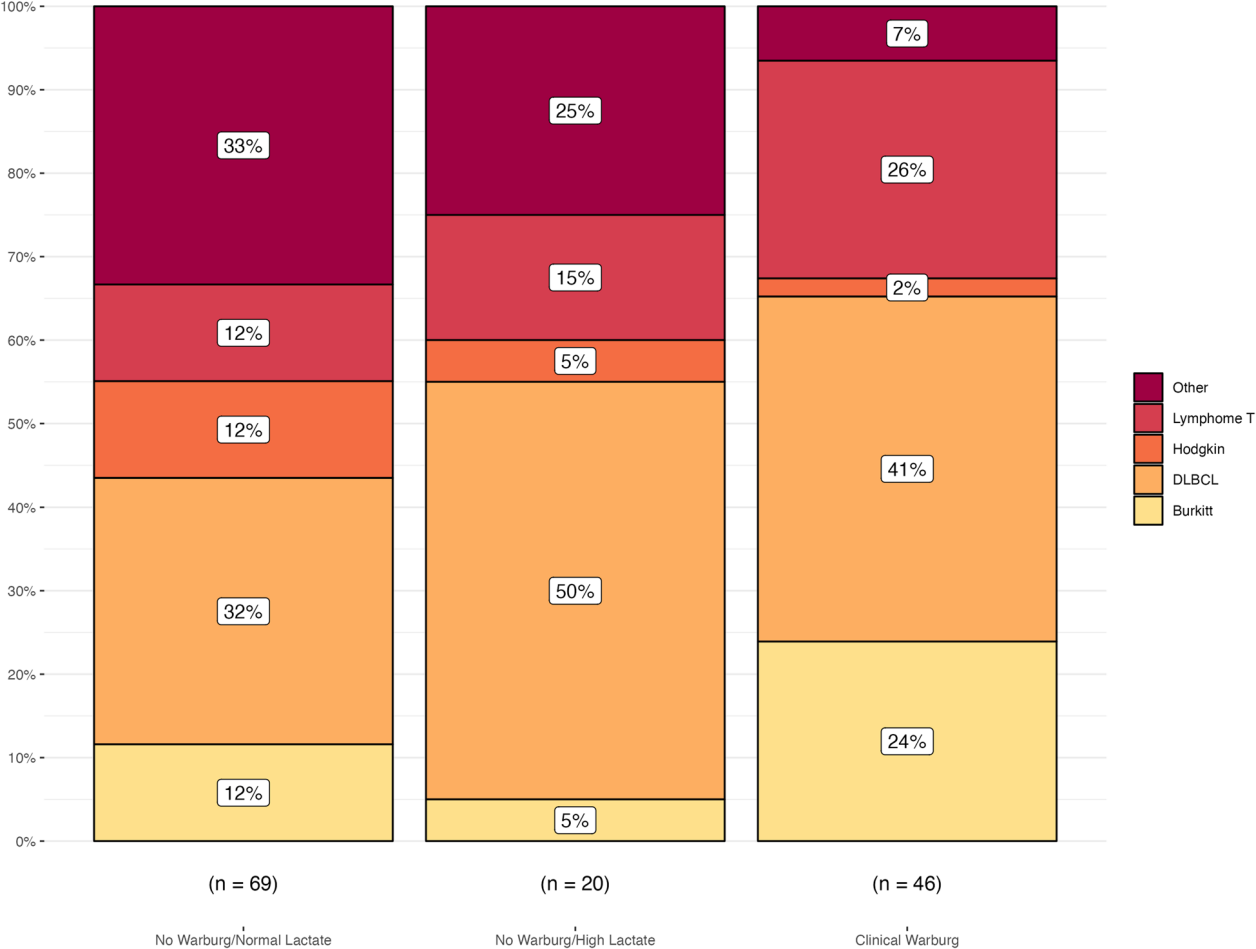
Distribution of hemopathy subtypes according to the Warburg category

The main reason for admission of CWE patients was the administration of a first-line chemotherapy with a high risk of complications in 30% of cases, followed by tumor lysis syndrome (20%) and acute respiratory distress (17%). The comparative baseline characteristics of the three groups are reported in Table 1. The three groups were similar in terms of sex and age. However, the distribution of lymphoma subtypes differed between the groups, with the CWE group having a higher proportion of patients with Burkitt lymphoma (24%) and T cell lymphoma (26%) compared to the other two groups. The most represented aggressive lymphoproliferation in the overall population was diffuse large B cell lymphoma (DLBCL), and the prevalence was higher in the CWE and NW-HL groups than in the NW-NL group (41% and 50% versus 32%, respectively) (Fig. 3). Tumor burden was greater in the CWE group, as indicated by higher serum LDH levels with a median of 2422 UI/L compared to 890 and 828 UI/L in the NW-HL and NW-NL groups, respectively. This was also reflected by the higher incidence of tumor lysis syndrome in CWE patients (54% vs. 40% and 32% in the NW-HL and NW-NL groups, respectively). The subset of patients with HLH syndrome was underrepresented in the CWE group (11%). However, 6 patients with HLH were excluded from the diagnosis of CWE due to the presence of shock and therefore categorized as NW-HL. Cerebral and bone involvements of the underlying hematological malignancy were more frequent in CWE patients, involving 28% and 24% of patients in the CWE group versus 15% and 10% in the NW-HL group, respectively (Additional file 1: Table S2). All patients with a CWE and non-Hodgkin B cell lymphoma with bone localization exhibited a c-MYC mutation. Hypercalcemia was also found in 24% of cases in the CWE group compared to 15% and 8% in the NW-HL and NW-NL patients, respectively. Finally, CWE patients were more likely hypoglycemic (Table 1, Additional file 1: Figure S1).

**Table 1:** Baseline characteristics^a^

	No Warburg/normal lactate level (*n* = 69)	No Warburg/high lactate level (*n* = 20)	Clinical Warburg effect (*n* = 46)
Demographic characteristics			
Sex male—no. (%)	49 (71)	13 (65)	26 (58)
Age—year	58 (40–64)	61 (52–64)	60 (50–72)
Hemopathy characteristics			
Lymphoma type —no. (%)			
DLBCL^b^	22 (32)	10 (50)	19 (41)
Burkitt	8 (12)	1 (5)	11 (24)
Hodgkin	8 (12)	1 (5)	1 (2)
T cell lymphoma	8 (12)	3 (15)	12 (26)
Other	23 (33)	5 (25)	3 (7)
Inaugural malignancy—no. (%)	60 (87)	14 (70)	32 (70)
Stade IV^c^—no. (%)	63 (96)	20 (100)	45 (98)
EBV associated^d^—no. (%)	11 (18)	4 (20)	4 (9)
HIV positive—no. (%)	13 (19)	4 (20)	7 (15)
Serum LDH—UI/L	828 (433–1591)	890 (537–2066)	2422 (1099–4763)
Ki67—%	88 (80–95)	85 (80–90)	90 (90–100)
Associated hemophagocytic lymphohistiocytosis—no. (%)	18 (26)	6 (30)	5 (11)
Associated tumor lysis syndrome—no. (%)	22 (32)	8 (40)	25 (54)
Spontaneous tumor lysis syndrome—no. (%)	16 (23)	6 (30)	18 (39)
Clinical presentation at admission			
SAPS II	34 (23–51)	49 (40–59)	36.5 (28–49)
Total SOFA score at admission	3 (0–6)	6 (4–9)	2 (0–4)
Body temperature—°C	37.2 (36.7–37.8)	37.0 (36.2–37.4)	37.5 (36.8–38.0)
Mean blood pressure—mmHg	87 (78–100)	80 (72–89)	98 (87–106)
Heart rate—bpm	101 (88–110)	108 (103–121)	104 (91–120)
Respiratory rate—/min	22 (17–26)	24 (20–29)	23 (20–28)
Mottling skin—no. (%)	1 (1)	6 (30)	0 (0)
Metformin—no. (%)	4 (6)	5 (25)	0 (0)
Biological parameters at admission			
Serum lactate—mmol/L	1.5 (1.1–1.7)	5.1 (2.6–8.6)	4.4 (3.6–7.6)
Serum pH	7.42 (7.38–7.44)	7.38 (7.28–7.43)	7.40 (7.36–7.43)
PaCO_2_—mmHg	38 (34–43)	31 (26–39)	33 (29–38)
Serum bicarbonate—mmol/L	24 (22–29)	22 (17–24)	20 (18–23)
Glycemia—mmol/L	6.5 (5.9–7.3)	7.0 (6.0–8.4)	5.3 (3.7–6.2)
Hypoglycemia—no. (%)	6 (9)	3 (15)	26 (57)
PT—%	77 (64–92)	70 (55–83)	76 (66–85)
Factor V—%	124 (86–148)	100 (79–125)	130 (106–162)
Acute liver failure—no. (%)	2 (3)	3 (15)	0 (0)
Serum uric acid—mmol/L	387 (271–595)	357 (276–572)	551 (360–716)
Hypercalcemia—no. (%)	8 (12)	3 (15)	11 (24)
Serum albumin—g/L	31 (26–36)	31 (26–36)	33 (26–35)
Organ support at ICU admission			
Vasopressor—no. (%)	16 (23)	13 (65)	0 (0)
Mechanical ventilation—no. (%)	16 (23)	9 (45)	4 (9)
Renal replacement therapy—no. (%)	14 (20)	9 (45)	8 (17)

^a^Continuous values are reported as median (IQR)

^b^DLBCL refers to diffuse large B cell lymphoma

^c^According to the Ann Arbor classification

**Fig. 4:**
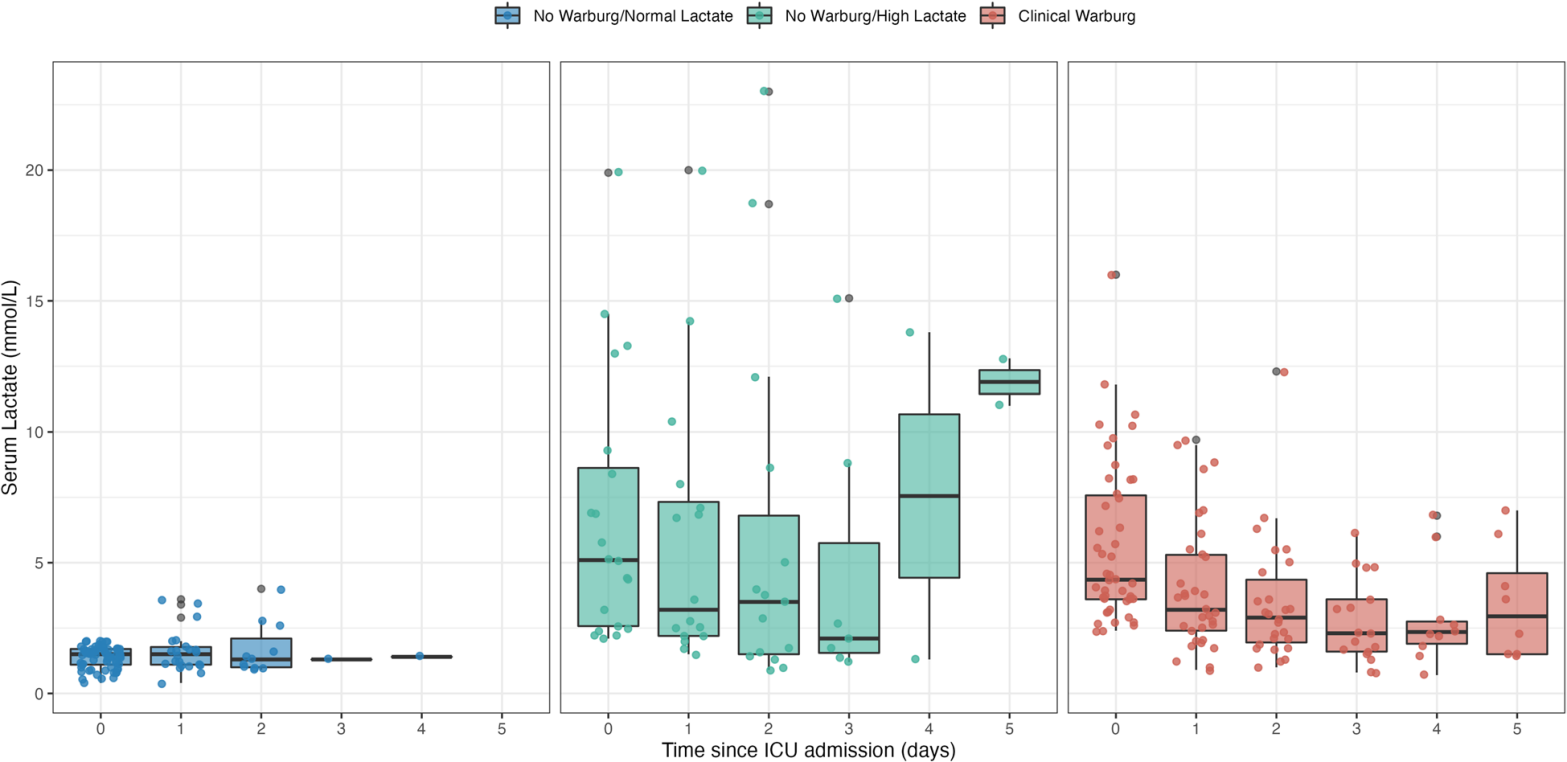
Serum lactate evolution according to the clinical Warburg group

The total SOFA score at admission was higher in the NW-HL group, with a median (IQR) of 6 (4–9) compared to 2 (0–4) for patients with a CWE. The mean arterial blood pressure was higher in CWE patients [98 mmHg (87–106)], with no patient requiring vasopressor support and none with mottled skin, while this was reported in 30% of cases in the NW-HL group. Otherwise, the heart and respiratory rates, as well as body temperature, did not differ significantly. Despite a lower SOFA score, the lactate levels at baseline were similar between CWE and NW-HL patients, with a median of 4.4 mmol/L (3.6–7.6) and 5.1 mmol/L (2.6–8.6), respectively (Table 1). The serum lactate trajectories within the days following ICU admission were similar for patients presenting a CWE, with a significant drop on day one (Fig. 4). Upon admission, blood gases displayed decompensated metabolic acidosis in the NW-HL group, while it was compensated in the CWE group and normal in the NW-NL group (Table 1). Finally, the CWE group had a higher frequency of patients who did not require organ support, with no patient requiring vasopressors, only 9% requiring mechanical ventilation and 17% requiring renal replacement therapy.

### Risk factors associated with 1-year mortality

**Fig. 5:**
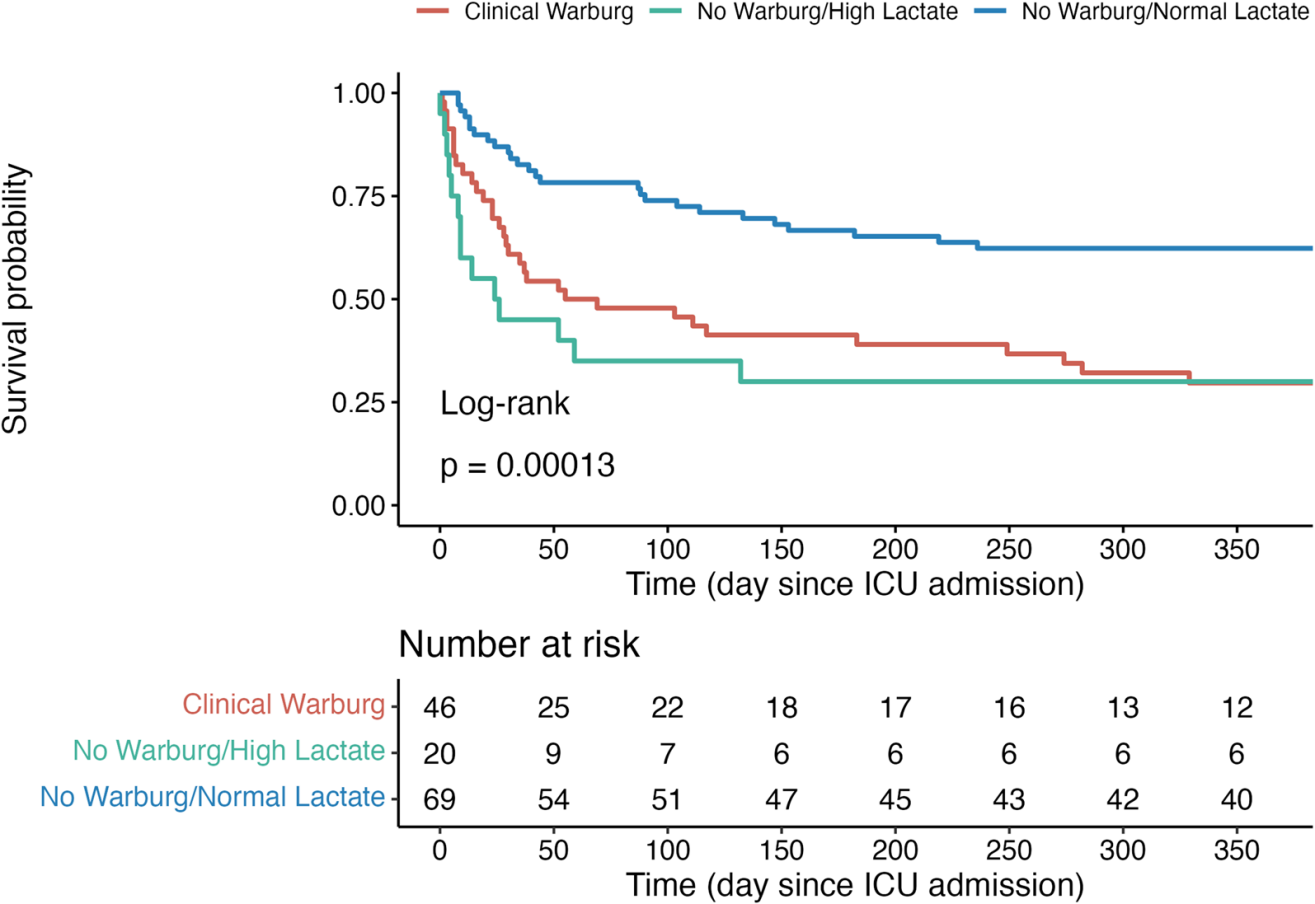
Kaplan–Meier survival estimates according to the Warburg group

At 1 year, 72 patients (53%) had died. There was a noticeable difference in the distribution of deaths among the three groups. Specifically, 70% of the patients in both the CWE and NW-HL groups died at 1 year, in contrast to the NW-NL group, which had a 1-year mortality rate of 38% (Fig. 5, Additional file 1: Figure S2). Factors independently associated with 1-year mortality by multivariable Cox survival analysis were the age [HR = 1.02 CI 95% (1.00–1.04)], total SOFA score at admission [HR = 1.19 CI 95% (1.12–1.25)] and both CWE and NW-HL groups [HR = 3.87 CI 95% (2.13–7.02) and HR = 3.34 CI 95% (1.68–6.60)] (Table 2**, **Additional file 1: Table S3, S4).

**Table 2:** Covariates associated with death at 12 months by Cox survival analysis

	Unadjusted analysis		Multivariable analysis	
	HR	95% CI	HR	95% CI
No Warburg/normal lactate level	Reference	Reference	Reference	Reference
Clinical Warburg	2.50	(1.49–4.21)	3.87	(2.13–7.02)
No Warburg/high lactate level	3.15	(1.64–6.04)	3.34	(1.68–6.60)
Tumor lysis syndrome	1.49	(0.94–2.37)	–	–
Spontaneous tumor lysis syndrome	1.88	(1.17–3.02)	1.53	(0.94–2.47)
Inaugural malignancy	0.54	(0.32–0.89)	–	–
Total SOFA score at admission	1.13	(1.08–1.17)	1.19	(1.12–1.25)
Age	1.02	(1.01–1.04)	1.02	(1.00–1.04)
Hemophagocytic lymphohistiocytosis	1.72	(1.03–2.89)	–	–
SAPS II at admission	1.03	(1.02–1.04)	–	–
Gynecological localization	3.08	(0.96–9.86)	–	–

### Factors associated with a clinical Warburg effect

By multivariable analysis, the main factors associated with a CWE were the presence of tumor lysis syndrome [OR = 2.84 CI 95% (1.14–7.42)], bone involvement of the underlying malignancy [OR = 3.58 CI 95% (1.02–12.91)], the total SOFA score at admission [OR = 0.81 CI 95% (0.69–0.91)] and hypoglycemia at admission [OR = 14.90 CI 95% (5.42–47.18)] (Table 3).

**Table 3:** Covariates associates with a clinical Warburg effect by logistic regression

	Unadjusted analysis		Multivariable analysis	
	OR	95% CI	OR	95% CI
Inaugural hemopathy	0.46	(0.20–1.08)	0.41	(0.14–1.21)
Bone localization	3.18	(1.19–8.88)	3.58	(1.02–12.91)
Tumor lysis syndrome	2.34	(1.14–4.89)	2.84	(1.14–7.42)
Central nervous system localization	2.79	(1.14–6.99)	–	–
Total SOFA score at admission	0.84	(0.74–0.94)	0.81	(0.69–0.91)
Hypoglycemia	11.56	(4.85–29.87)	14.90	(5.42–47.18)

### Robustness of findings

Bootstrapping and overlap propensity score weighted analyses were in line with mortality results suggesting that the Clinical Warburg status association remains robust in our cohort. The results are detailed in the supplemental data (Additional file 1: Table S5, Figure S3–S5).

## Discussion

This is the first and largest study describing the clinical characteristics and long-term outcomes of patients admitted to the ICU with lymphoma and a CWE. We defined three homogeneous groups based on outcomes and clinical characteristics. Moreover, we found that a CWE was associated with a high tumor burden. Finally, we found that CWE was highly associated with 1-year mortality. In most cases, these patients were discharged alive from the ICU and then died within the following year. Interestingly, we found that CWE and NW-HL groups had similar 1-year mortality rates.

The bulk of clinical evidence regarding this condition is derived from case reports [14, 15, 31, 32]. Similar to our study, these reports demonstrated a decrease in lactate levels after the initiation of chemotherapy, with normalization occurring within a week. The mortality among these patients was high and consistent with our findings emphasizing the urgent need to consider this cluster of patients as an oncological emergency. As previously contended by Otto H. Warburg, this condition may be associated with malignant progression and resistance to therapy [33].

Although lactate has primarily been considered as a biomarker, its biological effects should not be underestimated. Indeed, it is an oncometabolite involved in all main steps of malignant progression through several mechanisms [33–38]. Indeed, lactate is a key factor in tumor growth, promotes angiogenesis and cell migration by stimulating vascular endothelial growth factor (VEGF) expression on endothelial cells and enhances tumor immune escape [34]. However, while the lactate production by tumor cells itself may be harmful, the limited number of cases that reported the use of renal replacement therapy do not provide unequivocal support for this hypothesis [16, 39]. Based on this assumption, some authors have advocated the use of bicarbonate as anticancer adjuvant therapy in this subgroup of patients [40]. Most of these reports pertain to patients with solid tumors and have yielded mixed results. Owing to the close connection between CWE and tumor lysis syndrome in the lymphoma population on the one hand and the potentially detrimental effect of alkaline therapy in tumor lysis syndrome on the other, this treatment would be best tested in the setting of a trial [41]. Moreover, we have no evidence that influencing circulating lactate levels and pH positively impacts the lactate production in the tumoral microenvironment. Indeed, the CWE leads to a unique metabolic state where the cytosolic lactate is mainly exported through up-regulated lactate-proton symporter (MCTs) [42, 43] implying the participation of Na^+^/H^+^ exchangers [44], vacuolar H^+^-ATPases [45], and carbonic anhydrases which hydrate CO_2_ from oxidative metabolism to form H^+^ and bicarbonate [46]. These intricate mechanisms result in a specific acid–base metabolic microenvironment characterized by an alkaline intracellular pH and an acidic extracellular environment [43, 46]. Non-tumoral cells exposed to such chronic acidic conditions are then able to induce renal compensation, which could explain why CWE patients were more likely to maintain a normal pH despite minimal respiratory compensation and had higher serum bicarbonate levels than expected.

Some authors also considered the administration of thiamine in adjunction to chemotherapy [17, 18, 47, 48]. Indeed, thiamine deficiency has been reported to be associated with the CWE genesis. Thiamine is a cofactor in the conversion of pyruvate to acetyl-CoA through the action of pyruvate dehydrogenase. A deficiency in this cofactor leads to a pyruvate shunt through the glycolytic pathway causing lactate production. If the exact prevalence of thiamine deficiency among CWE patients is yet to be investigated, it could be contemplated as a safe, low-cost adjunctive therapy in this context.

Among several factors, the bone localization of the underlying malignancy was found to be independently associated with the development of CWE. Our primary hypothesis to explain this observation is that an interaction between tumor cells and the bone microenvironment may drive the glycolytic phenotype. Indeed, Diedrich et al. demonstrated that bone marrow adipocytes can enhance the glycolysis phenotype in metastatic prostate tumors through HIF-1α [49]. Additionally, other authors have reported the implication of aerobic glycolysis in osteosarcoma, particularly through c-MYC [50, 51]. In our cohort, all patients with CWE and non-Hodgkin B cell lymphoma with bone localization had a c-MYC mutation which may support this hypothesis. However, further exploration is needed to confirm this relationship.

Approximately half of CWE patients were hypoglycemic at the time of ICU admission, a phenomenon previously reported in several case reports and referred to as “Hyper-Warburgism” [52, 53]. Although the underlying mechanisms remain unclear, several hypotheses can be proposed to explain this feature. First, mutations in isocitrate dehydrogenase 2 (IDH-2) have been linked to the stabilization of HIF-1 and enhanced glucose uptake in in vitro studies [54]. However, IDH-2 mutation is more commonly associated with angioimmunoblastic T cell lymphomas [55], which were underrepresented in our cohort. More interestingly, c-MYC and p53 mutations can induce the overexpression of glucose transporters (GLUT), possibly contributing to the clinical hypoglycemia observed in our population [54, 56]. A detailed analysis of the glucose course, immunohistological characteristics, and long-term outcomes of these patients deserves further investigation in a larger cohort.

By identifying the specific subgroup of patients with CWE admitted to the ICU with a lymphoproliferative diagnosis, we highlighted the significant prevalence of this underdiagnosed phenomenon. This offers to the clinician an early biomarker of the tumor burden. Despite the absence of advanced organ support, their long-term outcome is similar to patients with lymphoproliferation associated with shock. Therefore, the emergency management of these patients requires specialized care from both hematological and ICU teams. The association with tumor lysis syndrome also deserves to be highlighted, placing these patients in the high-risk category of metabolic complications.

Our study has several strengths. First, our extensive screening method allowed us to present an accurate proportion of patients presenting this condition. Additionally, as all patients were treated in our center, we obtained a complete follow-up for our primary outcome and reported the mortality rate with greater precision. Finally, thanks to our center's extensive experience in managing these patients, we were able to provide a comprehensive understanding of this population. This study provides useful red flags for identifying these patients as a specific subgroup of cancer patients and as a starting point for designing future interventional studies to improve their prognosis. However, the results presented must be interpreted in light of the study’s limitations. First, all concerns related to the study’s retrospective design apply here, and even sensitivity analyses may not account for potential unmeasured confounding variables. Furthermore, this study presents a homogeneous cohort from a single-center highly specialized in hematological malignancies, which may limit the generalizability of the findings to other settings. In addition, multiple case reports have highlighted liver dysfunction related to lymphoproliferative disorders [57]. Although the incidence of this phenomenon has never been reported, its main mechanisms are direct infiltration, bile duct obstructions from enlarged lymph nodes, and paraneoplastic syndromes (e.g., vanishing bile duct syndrome). Therefore, it is possible that some patients with a degree of partial liver insufficiency were classified as clinical Warburg. Finally, we had to contend with the absence of a previous clinical definition of this phenomenon, which may have affected the relevance of our case selection. Serum lactate levels can be elevated by other circumstances, such as septic shock. Thus, we may have misclassified patients with a concomitant CWE and shock. However, as previously described by Hourmant et al. the prognosis of these patients is mainly driven by organ failures [27] and differentiating patients with a Warburg effect among this population may be clinically irrelevant.

## Conclusion

CWE is associated with a higher tumor burden and worse 1-year mortality compared to patients without this condition. When clinicians identify such patients, they should be wary of the urgency of their management, as their outcomes are comparable to those with lymphoma and shock, even when no advanced organ support is needed. Further studies are required to confirm and expand upon these findings.

### Take-home messages:

- CWE as an early biomarker: Identifying CWE in ICU patients can function as an early biomarker for assessing tumor burden. Moreover, it is associated with poor long-term outcomes.
- Specialized emergency care needed: Managing patients with CWE requires specialized care involving collaboration between hematological and ICU teams. Timely initiation of chemotherapy discussions is crucial in their emergency management.
- Consider thiamine supplementation: Consider proposing thiamine supplementation for patients diagnosed with CWE.

### Supplementary Information

**Additional file 1: Figure S1.** Blood glucose levels at admission according to the Clinical Warburg group. **Figure S2.** Death at 12 months mortality proportions distribution according to the Clinical Warburg group4. **Figure S3.** Bootstrap sensitivity analysis of the Hazard ratio estimation according to the Clinical Warburg status4. **Figure S4.** Distribution balance of propensity score5. **Figure S5.** Kaplan–Meier survival estimates according to the Warburg group after propensity weighting5. **Table S1.** Baseline characteristics and outcomes of patients excluded due to the absence of serum lactate measurement6. **Table S2.** Documented localization of the hemopathy^a^7. **Table S3.** Covariates associated with death at 12 months by unadjusted Cox survival analysis8. **Table S4.** Covariates associated with death at 12 months by Cox survival analysis (Model 2)9. **Table S5.** Average Treatment effect on the Treated (ATO) and Odds Ratio (OR) after overlap propensity score 10.

## Data Availability

Study protocol, data set and statistical code are available from the corresponding author on reasonable request.

## Abbreviations

CWE: Clinical Warburg effect
HLH: Hemophagocytic lymphohistiocytosis
ICU: Intensive care unit
NW-HL: No Warburg with high lactate level
NW-NL: No Warburg with no lactate elevation
SOFA: Sequential Organ Failure Assessment
SAPS II: Simplified Acute Physiology Score
ATO: Average treatment effect on the treated
TLS: Tumor lysis syndrome
