# Supplementary material for: Clinical Warburg effect in lymphoma patients admitted to intensive care unit
Source: Ann Intensive Care. 2023 Oct 5;13:97. doi: 10.1186/s13613-023-01192-z (PMC10555986; doi:10.1186/s13613-023-01192-z)
Supplement: Supplementary file 1 — Additional file 1: Figure S1. Blood glucose levels at admission according to the Clinical Warburg group. Figure S2. Death at 12 months mortality proportions distribution according to the Clinical Warburg group4. Figure S3. Bootstrap sensitivity analysis of the Hazard ratio estimation according to the Clinical Warburg status4. Figure S4. Distribution balance of propensity score5. Figure S5. Kaplan–Meier survival estimates according to the Warburg group after propensity weighting5. Table S1. Baseline characteristics and outcomes of patients excluded due to the absence of serum lactate measurement6. Table S2. Documented localization of the hemopathya7. Table S3. Covariates associated with death at 12 months by unadjusted Cox survival analysis8. Table S4. Covariates associated with death at 12 months by Cox survival analysis (Model 2)9. Table S5. Average Treatment effect on the Treated (ATO) and Odds Ratio (OR) after overlap propensity score 10. [file 13613_2023_1192_MOESM1_ESM.docx]

**Additional file**

Screening method ..................................................................................................................... 2

Statistical analysis details .......................................................................................................... 2

Figure S1: Blood glucose levels at admission according to the Clinical Warburg group…………………………………………………………………………………………3

[Figure S2: Death at 12 months mortality proportions distribution according to the Clinical Warburg group 4](#_Toc145183861)

[Figure S3. Bootstrap sensitivity analysis of the Hazard ratio estimation according to the Clinical Warburg status 4](#_Toc145183862)

[Figure S4. Distribution balance of propensity score 5](#_Toc145183863)

[Figure S5. Kaplan-Meier survival estimates according to the Warburg group after propensity weighting 5](#_Toc145183864)

[Table S1. Baseline characteristics and outcomes of patients excluded due to the absence of serum lactate measurement 6](#_Toc144475873)

[Table S2. Documented localization of the hemopathy^a^ 7](#_Toc144475874)

[Table S3. Covariates associated with death at 12 months by unadjusted Cox survival analysis 8](#_Toc144475875)

[Table S4. Covariates associated with death at 12 months by Cox survival analysis (Model 2) 9](#_Toc144475876)

[Table S5. Average Treatment effect on the Treated (ATO) and Odds Ratio (OR) after overlap propensity score 10](#_Toc144475877)

**Screening method**

We designed a program using Python 3.4 to extract from all medical records names, sex, hematologic diagnosis, and basic clinical and laboratory data. For patients with aggressive lymphoproliferative disease, we double-checked the diagnosis and all the other data extracted before filling out the case report form. After screening, we excluded patients who had already received a first administration of chemotherapy ≥72h prior to ICU admission related to the current lymphoproliferation diagnosis. Clinical, biological, and imaging data were retrieved from medical records. Outcomes were also collected retrospectively from electronic medical records.

**Statistical analysis details**

Multivariable models were built using a conditional backward stepwise variable selection process based on variable influence in univariate analysis. Critical entry and exit p values were 0.2 and 0.1, respectively. Correlation and interaction were carefully checked within the final models as well as assumptions for the log-linearity of continuous variables and proportional hazard assumptions for survival models. According to the model, data are given as odds ratios (OR, 95% CI) or hazard ratios (HR, 95% CI). To further assess the influence of CWE on mortality, we performed an overlap propensity score weighting analysis. Absolute standardized differences (ASDs) were calculated for each variable according to the CWE diagnosis. An ASD >0.1 was considered a significant imbalance. We assessed the propensity score distribution before and after weighting to check its quality and the influence of CWE on death at one year was then estimated by the average treatment effect on the treated (ATO) and OR. We also performed as sensitivity analysis, a complex bootstrap resampling. First, the bootstrapping technique resampled the original set 10 000 times with replacement. Then, in each set, we assessed the unadjusted risk for mortality. Statistical significance was considered using two-sided tests with a critical alpha risk of 0.05. Statistical analyses were performed using R version 4.2.2 (R Foundation for Statistical Computing) with the packages' dpylr,' 'ggplot2,' 'ggstatplot',' survey,' 'survival,' 'tableone' and 'PSweight.'

Figure S1: Blood glucose levels at admission according to the Clinical Warburg group


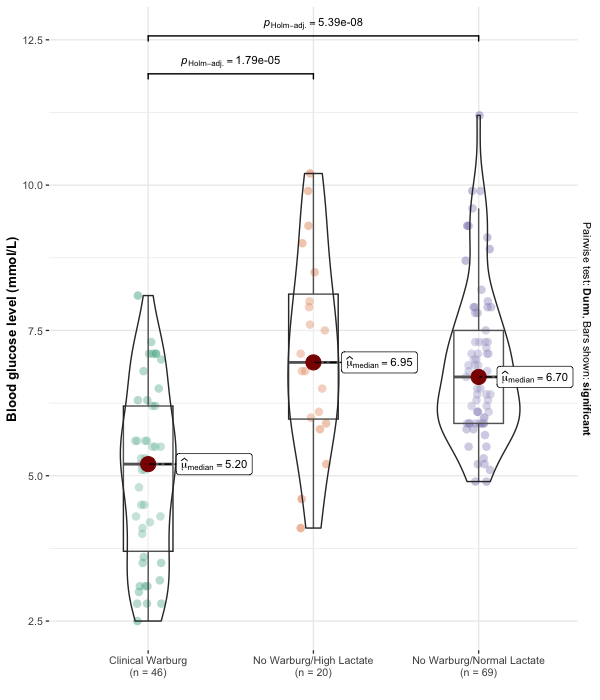


Figure S2: Death at 12 months mortality proportions distribution according to the Clinical Warburg group


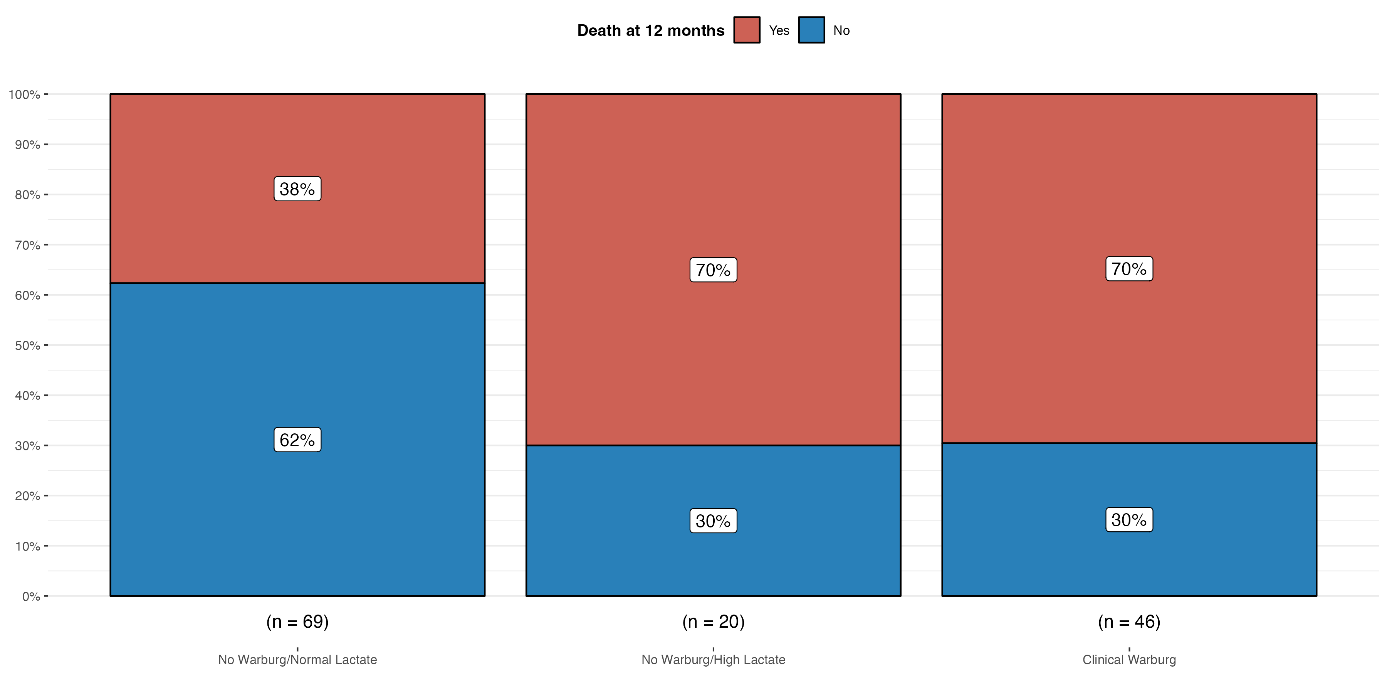


Figure S3. Bootstrap sensitivity analysis of the Hazard ratio estimation according to the Clinical Warburg status


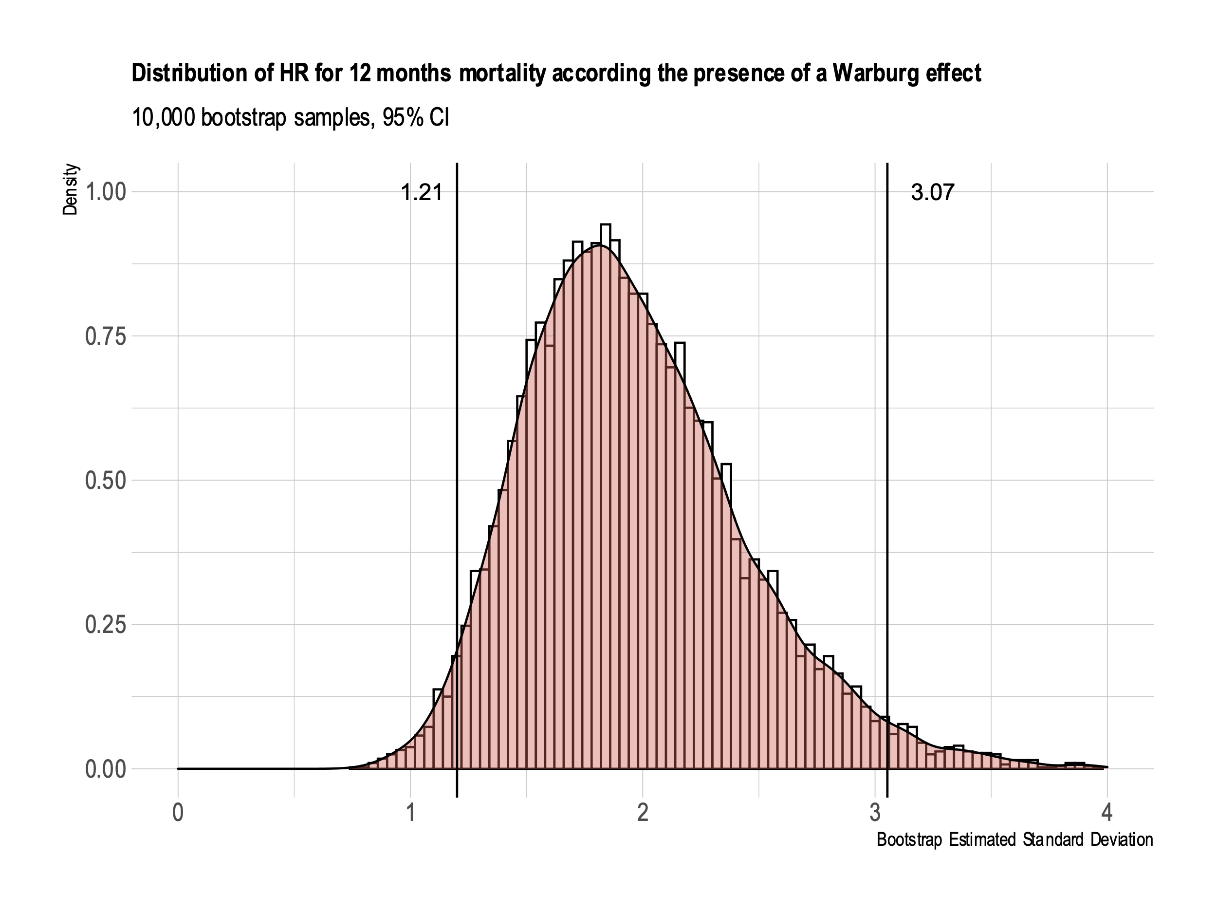


Figure S4. Distribution balance of propensity score


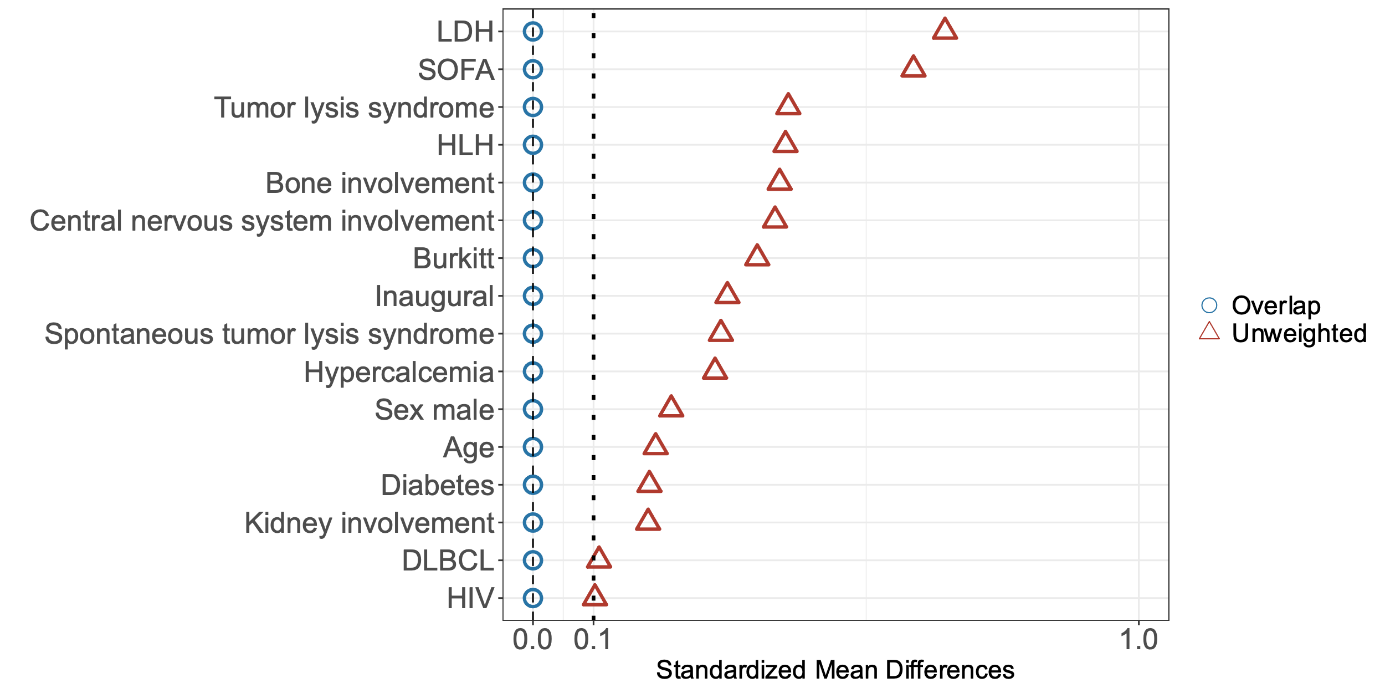


Figure S5. Kaplan-Meier survival estimates according to the Warburg group after propensity weighting


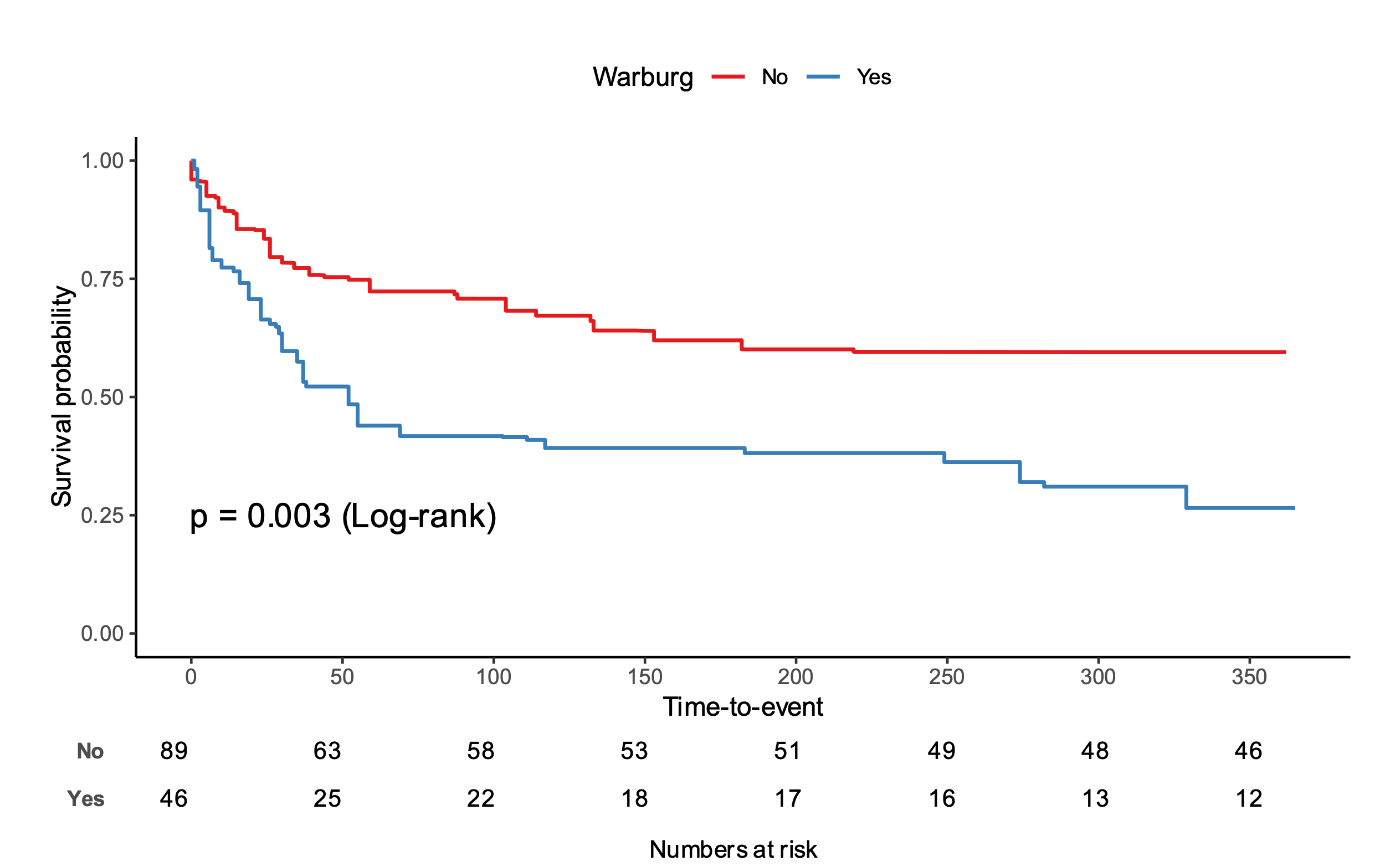


Table S1. Baseline characteristics and outcomes of patients excluded due to the absence of serum lactate measurement**^a^**

|  | Excluded due to no lactate measurement  (n = 23) |
| --- | --- |
| **Demographic characteristics** |  |
| Sex male —no. (%) | 15 (65) |
| Age — year | 49 (31-69) |
|  |  |
| **Hemopathy characteristics** |  |
| Lymphoma type —no. (%) |  |
| DLBCL^b^ | 13 (59) |
| Burkitt | 3 (14) |
| Hodgkin | 0 (0) |
| T Cell Lymphoma | 3 (14) |
| Other | 3 (14) |
| Inaugural malignancy —no. (%) | 21 (91) |
| Stade IV^c^—no. (%) | 17 (74) |
| EBV associated^d^ —no. (%) | 5 (22) |
| HIV positive —no. (%) | 1 (4) |
| Serum LDH — UI/L | 904 (442-1395) |
| Ki67 — % | 90 (80-95) |
| Associated Hemophagocytic Lymphohistiocytosis —no. (%) | 1 (4) |
| Associated Tumor Lysis Syndrome —no. (%) | 7 (30) |
| Spontaneous Tumor Lysis Syndrome —no. (%) | 4 (17) |
|  |  |
| **Clinical presentation at admission** |  |
| IGS2 Score | 30 (19-35) |
| Total SOFA Score | 1 (0-2.5) |
| Body temperature — °C | 37.0 (36.8-37.2) |
| Mean blood pressure — mmHg | 92 (86-101) |
| Heart rate — bpm | 98 (79-103) |
| Respiratory rate — /min | 20 (18-25) |
| Mottling skin —no. (%) | 0 (0) |
| Metformin —no. (%) | 0 (0) |
|  |  |
| **Biological parameters at admission** |  |
| Serum Bicarbonate — mmol/L | 22 (20-23) |
| PT — % | 80 (71-91) |
| Factor V — % | 121 (85-134) |
| Acute liver failure —no. (%) | 1 (4) |
| Serum uric acid — mmol/L | 386 (293-452) |
| Hypercalcemia —no. (%) | 3 (13) |
| Serum albumin — g/L | 37 (34-39) |
|  |  |
| **Organ support at ICU admission** |  |
| Vasopressor —no. (%) | 0 (0) |
| Mechanical ventilation —no. (%) | 1 (4) |
| Renal replacement therapy —no. (%) | 1 (4) |
|  |  |
| **Outcomes** |  |
| ICU death —no. (%) | 1 (4) |
| Death at 12 months —no. (%) | 9 (39) |

^a^Continuous values are reported as median (IQR)

Table S2. Documented localization of the hemopathy^a^

|  | No Warburg/Normal Lactate level  (n = 69) | No Warburg/High Lactate level  (n = 20) | Clinical Warburg Effect  (n = 46) |
| --- | --- | --- | --- |
| Adenopathy | 60 (87) | 17 (85) | 42 (91) |
| Bone marrow | 17 (25) | 6 (30) | 12 (26) |
| Central Nervous System | 8 (12) | 3 (15) | 13 (28) |
| Bone | 6 (9) | 2 (10) | 11 (24) |
| Liver | 16 (23) | 6 (30) | 10 (22) |
| Spleen | 13 (19) | 6 (30) | 8 (17) |
| Gastrointestinal | 9 (13) | 6 (30) | 8 (17) |
| Kidney | 4 (6) | 1 (5) | 5 (11) |
| Pleural | 9 (13) | 2 (10) | 7 (15) |
| Adrenal gland | 0 (0) | 0 (0) | 3 (7) |
| Gynaecologic | 1 (1) | 0 (0) | 2 (4) |
| Blood | 1 (1) | 2 (10) | 3 (7) |
| ORL | 4 (6) | 4 (20) | 1 (2) |
| Urological | 2 (3) | 3 (15) | 0 (0) |
| Heart | 8 (12) | 0 (0) | 3 (7) |
| Skin | 2 (3) | 2 (10) | 2 (4) |
| Mediastinum | 9 (13) | 0 (0) | 4 (9) |
| Lung | 5 (6) | 0 (0) | 2 (4) |
| Other | 1 (1) | 0 (0) | 1 (2) |
| ^a^Results are presented as no. (%) | | | |

Table S3. Covariates associated with death at 12 months by unadjusted Cox survival analysis

|  | HR | 95% CI |
| --- | --- | --- |
| No Warburg/Normal Lactate level | Reference | Reference |
| Clinical Warburg | 2.50 | (1.49-4.21) |
| No Warburg/High Lactate level | 3.15 | (1.64-6.04) |
| Tumor lysis syndrome | 1.49 | (0.94-2.37) |
| Spontaneous tumor lysis syndrome | 1.88 | (1.17-3.02) |
| Inaugural malignancy | 0.54 | (0.32-0.89) |
| Total SOFA score at admission | 1.13 | (1.08-1.17) |
| Age | 1.02 | (1.01-1.04) |
| Hemophagocytic lymphohistiocytosis | 1.72 | (1.03-2.89) |
| SAPS II at admission | 1.03 | (1.02-1.04) |
|  |  |  |
| LDH — per 1000 UI/L | 1.06 | (0.97-1.15) |
| EBV status | 0.85 | (0.42-1.71) |
| Inaugural status | 0.54 | (0.32-0.89) |
| HIV | 1.03 | (0.58-1.85) |
| Hypercalcemia | 0.98 | (0.53-1.81) |
| Diabetes | 1.23 | (0.68-2.20) |
| Chronic kidney disease | 1.33 | (0.64-2.79) |
| IGS2 score at admission | 1.03 | (1.02-1.04) |
| Serum albumin | 0.99 | (0.95-1.02) |
| Localization |  |  |
| Gynecological | 3.08 | (0.96-9.86) |
| Bone marrow | 0.85 | (0.49-1.46) |
| Liver | 0.88 | (0.50-1.53) |
| Spleen | 1.24 | (0.71-2.16) |
| Gut | 0.76 | (0.39-1.49) |
| Kidney | 0.85 | (0.34-2.12) |
| Bone | 1.15 | (0.61-2.19) |
| Pleural | 1.18 | (0.62-2.25) |
| Adrenal | 1.87 | (0.46-7.64) |
| Blood | 1.82 | (0.73-4.53) |
| Ear, nose and throat | 1.87 | (0.86-4.09) |
| Urological | 0.3 | (0.04-2.19) |
| Heart | 0.42 | (0.13-1.35) |
| Skin | 1.88 | (0.76-4.69) |
| Mediastinum | 0.68 | (0.28-1.70) |
| Lung | 1.02 | (0.32-3.24) |

Table S4. Covariates associated with death at 12 months by Cox survival analysis (Model 2)

|  | Unadjusted analysis | |  | Multivariable analysis | |
| --- | --- | --- | --- | --- | --- |
|  | HR | 95% CI |  | HR | 95% CI |
| No Warburg/Normal Lactate level | Reference | Reference |  | Reference | Reference |
| Clinical Warburg | 2.50 | (1.49-4.21) |  | 4.08 | (2.19-7.60) |
| No Warburg/High Lactate level | 3.15 | (1.64-6.04) |  | 3.27 | (1.65-6.50) |
| Tumor lysis syndrome | 1.49 | (0.94-2.37) |  | - | - |
| Spontaneous tumor lysis syndrome | 1.88 | (1.17-3.02) |  | 1.66 | (0.96-2.88) |
| Inaugural malignancy | 0.54 | (0.32-0.89) |  | - | - |
| Total SOFA score at admission | 1.13 | (1.08-1.17) |  | 1.19 | (1.12-1.25) |
| Age — per year | 1.02 | (1.01-1.04) |  | 1.02 | (1.00-1.04) |
| Hemophagocytic lymphohistiocytosis | 1.72 | (1.03-2.89) |  | - | - |
| SAPS II at admission | 1.03 | (1.02-1.04) |  | - | - |
| Gynecological localization | 3.08 | (0.96-9.86) |  | - | - |
| LDH — per 1000 UI/L | 1.06 | (0.97-1.15) |  | 0.97 | 0.86-1.08 |

Table S5. Average Treatment effect on the Treated (ATO) and Odds Ratio (OR) after overlap propensity score

|  | Estimate | Standard Error | 95% CI | P-value |
| --- | --- | --- | --- | --- |
| ATO | 0.32 | 0.09 | (0.15 - 0.48) | <0.001 |
| OR | 3.86 | 0.40 | (1.75 - 8.49) | <0.001 |
